# Genome-wide identification and expression analysis of the CONSTANS-like family in potato (*Solanum tuberosum* L.)

**DOI:** 10.3389/fgene.2024.1390411

**Published:** 2024-07-09

**Authors:** Wang Yin, Luo Wang, Qiqiong Shu, Mingjun Chen, Fei Li, Xiaobo Luo

**Affiliations:** ^1^ Guizhou Institute of Biotechnology, Guizhou Academy of Agricultural Sciences, Guiyang, China; ^2^ Ministry of Agriculture and Rural Affairs Key Laboratory of Crop Genetic Resources and Germplasm Innovation in Karst Region, Guiyang, China; ^3^ Guizhou Key Laboratory of Agriculture Biotechnology, Guiyang, China

**Keywords:** CONSTANS-like, gene family, tuberization, cold stress, qRT-PCR

## Abstract

The *CONSTANS-like* (*COL*) gene plays important roles in plant growth, development, and abiotic stress. A total of 15 *COL* genes are unevenly distributed on eight chromosomes in the potato genome. The amino acid length of the family members was 347–453 aa, the molecular weight was 38.65–49.92 kD, and the isoelectric point was 5.13–6.09. The *StCOL* family can be divided into three subfamilies by evolutionary tree analysis, with conserved motifs and similar gene structure positions in each subfamily. The analysis of promoter cis-acting elements showed 17 cis-acting elements related to plant hormones, stress, and light response. Collinearity analysis of *COL* genes of tomato, potato, and *Arabidopsis* showed that 13 *StCOL* genes in the different species may have a common ancestor. A total of 10 conserved motifs and six kinds of post-translational modifications in the 15 StCOL proteins were identified. The 15 *StCOL* genes exhibit a genomic structure consisting of exons and introns, typically ranging from two to four in number. The results showed that 10 genes displayed significant expression across all potato tissues, while the remaining five genes were down-expressed in potato transcriptome data. The q*uantitative* reverse transcription *polymerase chain reaction (*qRT-PCR) analysis exhibited differential expression of 8 *StCOL* genes in the potato leaves and tubers at different growth stages, as well as 7 *StCOL* genes under 2°C treatment conditions. These results suggested that the *StCOL* gene family may play an important role in regulating potato tuberization and responding to cold stress.

## 1 Introduction

*COL* is a zinc finger activator of transcription that contains two conserved elements in the N-terminal B-box domain and the C-terminal CCT domain (Imaizumi et al., 2005; Kikuchi et al., 2012). The *CO* (*CONSTANS*)/*COLs* can be divided into three major groups based on the divergence of conserved domains (Crocco and Botto, 2013). Group I members include two B-box domains and one CCT domain, while group II members possess one B-box domain and one CCT domain. Group III members contain one B-box and one diverged zinc finger structure (Li et al., 2020). *COL* genes have been reported in many plant species, such as *Arabidopsis* (Robson et al., 2001), rice (Griffiths et al., 2003), petunia (Khatun et al., 2021), and mango (Guo et al., 2022). *COL* genes, which are key genes in the photoperiodic pathway, play an important role in plant growth and development, regulating flowering, tuber formation, and abiotic stress (Turck et al., 2008; Kloosterman et al., 2013; Zhang et al., 2023).

Flowering is a vital growth transition period in plant growth and development and is one of the most important agronomic traits for crop yield (Steinbach, 2019). The promotion of flowering in *Arabidopsis* by *CO* is influenced by day length and serves as a central integrator in the photoperiodic flowering pathway (Samach et al., 2000). Previous studies have found that *CO* activates *SOC1* through *FT* to promote flowering in *Arabidopsis* (Yoo et al., 2005). *AtCOL1* and *AtCOL2* synergically regulate the circadian rhythm of *Arabidopsis* (Ledger et al., 2001). *AtCOL3* promotes lateral root development and aboveground branching, while *AtCOL4* promotes tolerance to abiotic stress (Datta et al., 2006; Min et al., 2015). Overexpression of *COL5* affects flowering time and the expression of *FT* and *SOC1* in *Arabidopsis* (Hassidim et al., 2009). Overexpression of *HvCO1* can accelerate the flowering time of wheat and mediate the upregulation of *HvFT1* under long-day (LD) conditions (Campoli et al., 2012). The grape gene *VvCO* plays a role in regulating the seasonal cycle of grape flowering. *VvCOL1* is mainly expressed in dormancy, and *CO* homologs of grapevine are also expressed in this unique organ (Almada et al., 2009). *GmCOL1a* and *GmCOL1b* function as flowering repressors in the photoperiodic flowering of soybean under long-day conditions (Cao et al., 2015). The overexpression of mango *MiCOL2A* and *MiCOL2B* significantly delayed flowering time in *Arabidopsis* under LD and SD conditions (Liang et al., 2023).

Cold stress is a major abiotic stress that adversely affects plant growth and crop productivity. Many studies have reported that *COL* genes play important roles in cold stress response (Mikkelsen and Thomashow, 2009; Jung et al., 2012; Huang et al., 2022). *AtCOL1* and *AtCOR27* are rapidly induced under cold stress by a CBF-independent pathway in *Arabidopsis* (Mikkelsen and Thomashow, 2009). *HIGH EXPRESSION OF OSMOTICALLY RESPONSIVE GENE 1* (*HOS1*) mediated *CO* degradation *via* a ubiquitin/proteasome pathway under cold stress (Jung et al., 2012). The *CO* transcript levels were increased at night by cooler temperature treatments (Kinmonth-Schultz et al., 2016). Five *COL* genes (*PaCOL2*, *PaCOL6*, *PaCOL8*, *PaCOL10,* and *PaCOL13*) were induced in response to low temperature in petunia (Khatun et al., 2021). A total of 10 *CaCOL* genes were identified in the pepper genome, five of which were significantly expressed under cold treatment (Huang et al., 2022).

Potato (*Solanum tuberosum* L.) is the world’s fourth largest food crop and is cultivated worldwide. Many studies have reported that *StCO* genes play an important role in potato tuber formation and flowering (González-Schain et al., 2012; Kloosterman et al., 2013; Abelenda et al., 2016). Potato *StCO* affects flowering and stem elongation and the autonomous pathway of potato tuber through different mechanisms (González-Schain and Suárez-López, 2008). Previous studies indicated that *StCO* was involved in the photoperiodic regulation of tuberization (González-Schain et al., 2012). It was found that *StCDF1* represses the transcription of *StCO1/2* under LD conditions in potato (Kloosterman et al., 2013). The silence of *StCOL1* was strongly associated with the downregulated expression of *StSP5G* in potato (Abelenda et al., 2016). The overexpression of *Nelumbo nucifera COL5* in potato increased the tuber weight and starch content under SD conditions (Cao et al., 2021). *StCDF1* is a non-redundant regulator of tuberization and binds to the promoter of *StCO1*, *StCO2,* and *StCO3* genes in potato (Ramírez and Gonzales, 2021). However, the molecular mechanism of *StCOL* genes in controlling tuber formation and clod stress response in potato remains unclear.

In this study, the conserved domains, evolutionary relationships, chromosome location, promoter element, and collinearity analysis of *StCOL* genes were systematically identified. The differential expression patterns of *StCOL* genes in different tissues and development stages were determined by potato transcriptome data and q*uantitative* reverse transcription *polymerase chain reaction* (qRT-PCR). The expression of *StCOL* genes at different treatment times under cold stress was also determined by qRT-PCR assays. The results will provide an important theoretical basis for exploring the molecular mechanism of potato tuberization and cold stress response.

## 2 Materials and methods

### 2.1 Identification of *COL* gene family members in potato

The conserved domain of COL proteins was downloaded from the Pfam database (http://pfam.xfam.org/family/PF06203 and PF00643) and was used as the template region to search *StCOL* sequences in the potato genome database (http://spuddb.uga.edu/). Hidden Markov model (HMM) software was used to select candidate *StCOL* genes, which were then submitted to NCBI (https://www.ncbi.nlm.nih.gov/) and UniProt (http://www.uniprot.org) for confirming the final *StCOL* gene family members.

### 2.2 Protein sequence and phylogenetic analysis of COLs

The online website cluster (https://pir.georgetown.edu/pirwww/search/multialn.shtml) was used to perform multiple alignments of the predicted protein sequences of the 15 *StCOLs* in potato, 17 *AtCOLs* in *Arabidopsis,* and 13 *SlCOLs* in tomato (Zobell et al., 2005). The unrooted phylogenetic tree was constructed using the MEGA 7 software with 1000 bootstrap replicates (Hall, 2013). The *StCOLs* were generated according to the phylogenetic relationships of 17 *AtCOL* genes with the *StCOL* gene and a specific gene nomenclature system.

### 2.3 Sequence analysis of *COL* genes in potato

The MEME (http://meme.sdsc.edu/meme/cgi-bin/meme.cgi) online software was used to assess the conservative area of the *StCOL* gene family protein sequence. The maximum ordinal number was set to 10, and the optimum motif width was 6–50 amino acid residues (Bailey et al., 2009). The gene structures of *StCOL*s were determined to obtain gene exons and containing substructures by Gene Structure Display Server (http://gsds.cbi.pku.edu.cn/). In addition, the cis-acting elements of *StCOL* promoter regions in potato were predicted by PlantCARE (PlantCARE/HTML/http://bioinformatics.psb.ugent.be/webtools/).

### 2.4 Synteny analysis and chromosomal localization of *COLs*

Collinearity analysis was conducted through PlantDGD software (http://pdgd.njau.edu.cn:8080/) (Qiao et al., 2019). The Multiple Collinearity Scan toolkit (MCScanX) was used to identify *StCOL* gene sequence repetition events. BLASTP was used to identify the species, symbiotic homologous pairs of the protein sequence. The protein sequence parameter was set to 1) alignment significance: e-value (default: 1 × 10^−5^), 2) MATCH_SCORE: final score (default: 50) (Wang et al., 2012). The obtained data were plotted by Circos online software (http://circos.ca/). Then, MapInspect software was used to locate *COL* genes whose relative positions have been identified in the potato genome database on potato chromosomes.

### 2.5 StCOL protein characterization

ExPASy online software (http://web.expasy.org/protparam/) was used to predict and analyze the amino acid number, theoretical molecular number, isoelectric point, and other physicochemical properties of the potato COL protein.

### 2.6 Expression analysis of *StCOL* genes

The publicly available RNA-seq data in the potato genome (http://spuddb.uga.edu/) were downloaded. The RNA-seq data of the root, tuber pith, tuber cortex, young tuber, mature tuber, shoot apex, tuber sprout, tuber peel, stamen, flower, petiole, leaf, stem, and stolon were selected to analyze the expression of *StCOL* genes. The “Normalized” function was used to normalize gene expression and construct the heat maps of *StCOL* gene expression using TBtools (Chen et al., 2020). The expression data are presented in Supplementary Table S1.

### 2.7 Total RNA extraction and qRT-PCR analysis

The leaves and tuber of the early maturing potato variety “Favorita” at three different growth stages (20, 42, and 65 days) were collected. The low-temperature sensitive variety “Desiree” was subjected to 2°C for 0 h, 1 h, 2 h, 24 h, and 48 h, and the leaves of all treatments were collected. The collected samples were stored in a −80°C refrigerator. The total RNA of all collected samples was isolated by a TaKaRa MiNiBEST Universal RNA Extraction Kit (TaKaRa, Beijing, China). Reverse transcription was performed with StarScript II RT Mix with gDNA Remover (GeneStar, Beijing, China). The PCR amplification was conducted on a CFX96 touch real-time PCR detection system (Bio-Rad, Hercules, CA, United States). β-actin was used as an internal reference gene. The Ct (2^−ΔΔCT^) method was used to calculate the relative expression of the *StCOL* genes (Livak and Schmittgen, 2001). The experiment was performed with three biological replicates. The primer sequences for qRT-PCR are listed in Supplementary Table S2. Duncan’s test with *p* < 0.05 was used to indicate significant differences.

## 3 Results

### 3.1 Identification of *COL* genes and multiple alignment analysis

A total of 15 potato *COL* genes were identified in the potato genome database. The genes were named *StCOL1*–*StCOL15*. The amino acid length of *StCOL* gene family members ranged from 347 to 453 aa, the molecular weight was 38.65–49.92 kD, and the isoelectric point was 5.13–6.09 (Supplementary Table S3). The isoelectric point of all 15 StCOL proteins was less than 7, the instability index was 6.94–59.96, and the aliphatic index ranged from 57.18 to 71.48. The grand average of hydropathicity ranged from −0.867 to −0.331, all of which were less than 0, indicating that StCOL proteins were hydrophilic.

### 3.2 Phylogenetic analysis of the *COLs*

**FIGURE 1:**
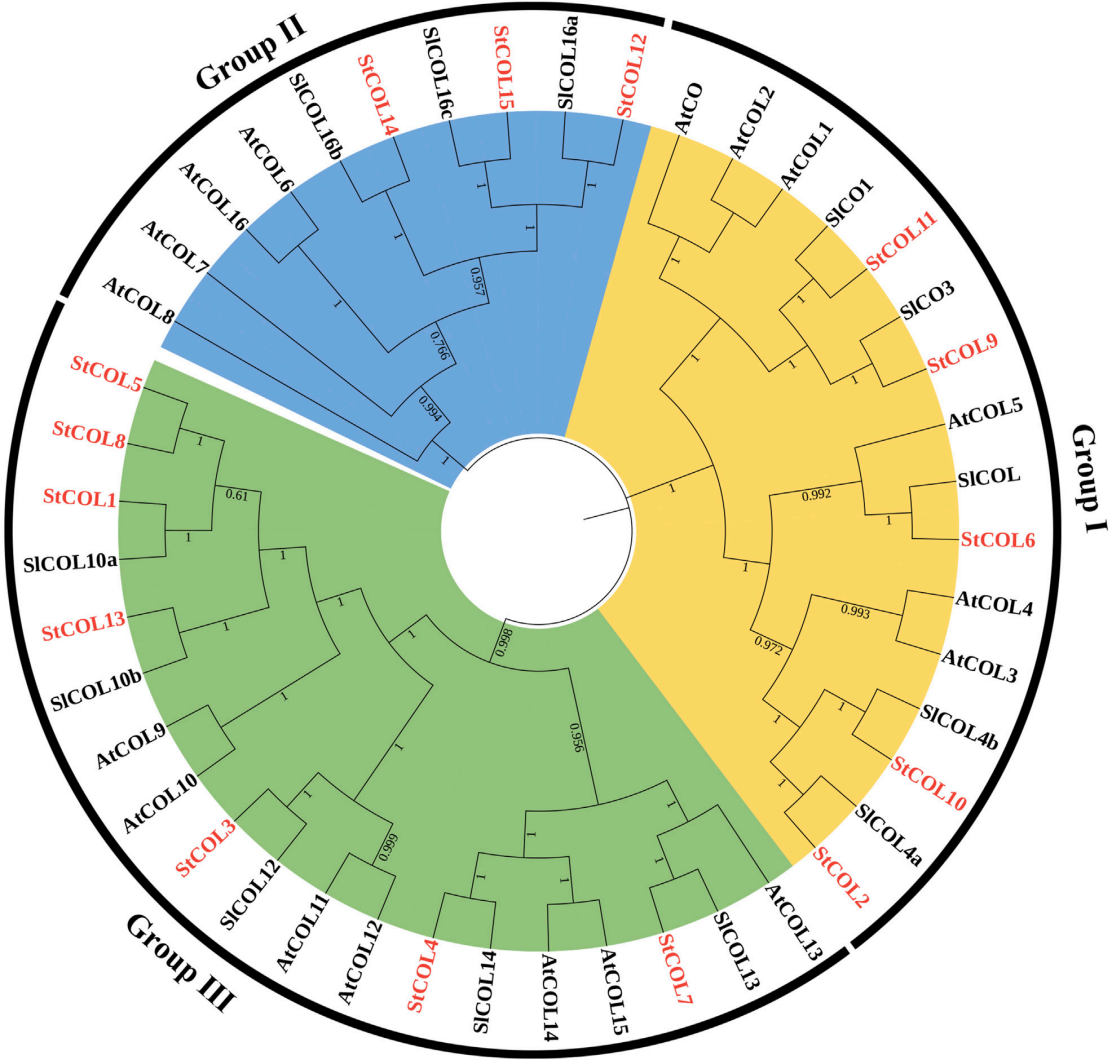
Neighbor-joining phylogenetic tree of COL proteins from *S. tuberosum* (St), *S. lycopersicum* (Sl), and *A. thaliana* (At). The classification of different groups is displayed with different colors.

In order to understand the evolutionary relationships among the *StCOL* gene family, *Arabidopsis,* and tomato, 15 *StCOLs*, 17 *AtCOLs*, and 13 *SlCOLs* were constructed by the phylogenetic tree (Figure 1). The StCOL protein family can be divided into three subfamilies: Group Ⅰ, Group Ⅱ, and Group Ⅲ. Group Ⅰ contained five *StCOLs*, six *AtCOLs*, and four *SlCOLs*. Group Ⅱ consists of three *StCOLs*, four *AtCOLs*, and three *SlCOLs*. Group III consists of seven *StCOLs*, seven *AtCOLs*, and five *SlCOLs*.

### 3.3 Gene structure and conserved motif analysis of *StCOLs*

**FIGURE 2:**
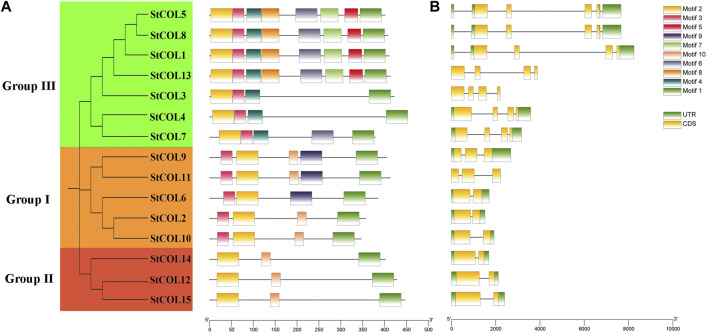
Gene structures and protein motifs of *StCOL* family genes. **(A)** The distribution of conserved motifs for StCOL genes. Different motifs and their relative positions are represented by the colored boxes. **(B)** Exon/intron structures of *StCOL* genes. Green boxes and yellow boxes represent coding sequence (CDS) and untranslated region (UTR), respectively.

A total of 10 conserved motifs were obtained by predicting the StCOL protein conserved motifs through the MEME website (Figure 2A). All 15 StCOL proteins contain motif 1 (CCT domain) and motif 2 (zinc finger B-box domain). The five *StCOLs* in group I all contain motif 1, motif 2, and motif 3. There is a motif 9 in *StCOL9* and *StCOL11*, but the location is slightly different. In group Ⅱ, *StCOL12*, *StCOL14*, and *StCOL15* all contain motif 1, motif 2, and motif 7. In group ⅠⅡ, all seven StCOL proteins contained motif 1, motif 2, motif 3, and motif 6, of which four StCOL proteins contained all eight motifs. In order to further understand the structural and conserved characteristics of the *StCOL* gene family, exons and introns of the *StCOL* gene family were analyzed. The 15 *StCOL* genes all contain between two and four exons and introns. The number of introns and exons of genes in group III and group Ⅱ was the same. The number of exons and introns varies in the different groups, which indicates that the functions of the different genes may be specific.

### 3.4 Promoter sequence analysis

**FIGURE 3:**
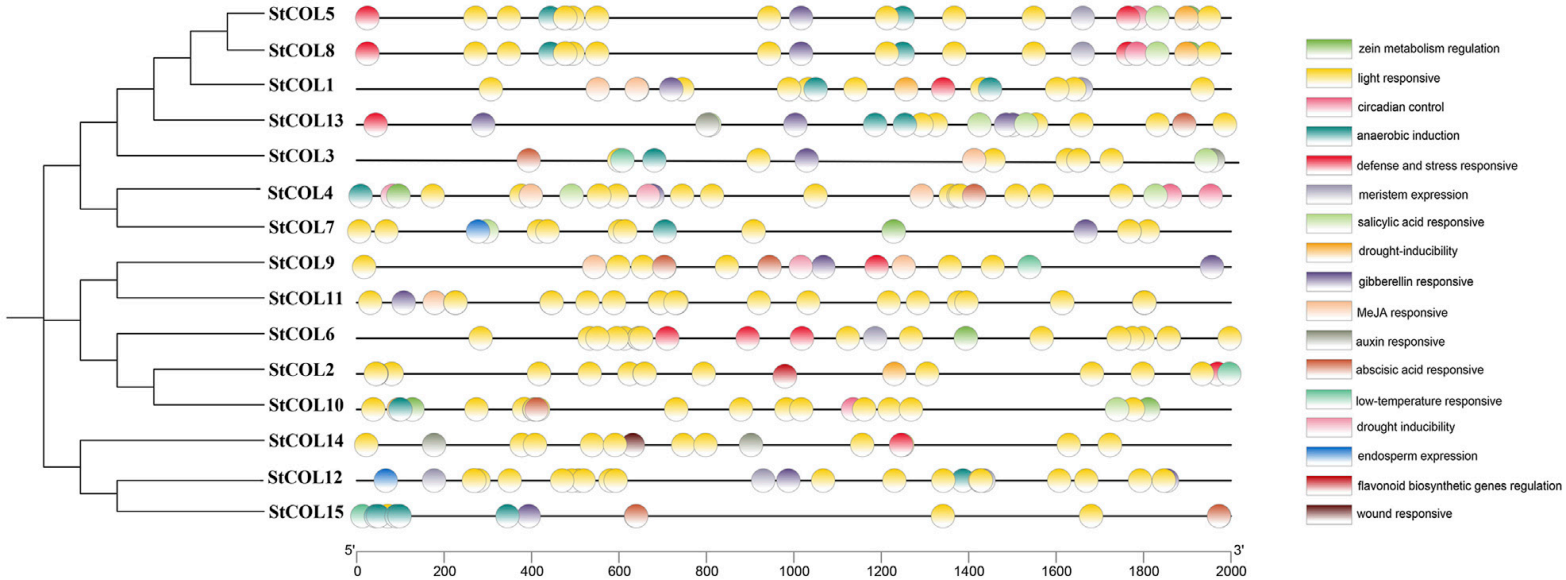
Cis-acting elements in the promoter region of *StCOL* genes.

Seventeen cis-acting elements related to stress response, growth and development, and hormone regulation were predicted in the potato *COL* gene family (Figure 3). Among them were five cis-acting elements related to growth and development, six cis-acting elements related to stress response, and six cis-acting elements related to hormone regulation response. Twelve genes contained response elements necessary for anerobic induction, 10 genes contained abscisic acid response elements, 11 genes contained gibberellin response elements, four genes contained cis-acting elements related to low-temperature stress response, and 15 *StCOL* contained light response elements. Most of the growth-related response elements are photoregulatory response elements. These results indicate that the *COL* gene may play an important role in anerobic induction, abscisic acid response, gibberellin response, low-temperature stress response, and light response conditions.

### 3.5 Prediction of protein sequence features

In order to understand the physicochemical properties of COL proteins in potato, the post-translational modifications were performed at ScanProsite (http://ca.expasy.org/tools/scanprosite/). It mainly includes six kinds of post-translational modifications in the 15 StCOL proteins, including phosphorylation sites protein kinase C (PKC), casein kinase II (CK II), CAMP-CGMP-kinase (cAMP-cGMP), tyrosine kinase (Tyr), N-myristoylation (N-Myr), and N-glycosylation (N-Glyc) (Supplementary Table S4). Each StCOL protein contains at least three and up to six post-translational modifications. The phosphorylation sites protein kinase C, casein kinase II, and N-myristoylation existed in 15 StCOL proteins. A total of eight StCOL proteins had cAMP-cGMP sites, five StCOL proteins with Tyr sites, and 13 StCOL proteins with N-Glyc sites were observed. The results suggested that StCOL proteins may exert their function through phosphorylation sites protein kinase C, casein kinase II, and N-myristoylation.

### 3.6 Chromosome location and duplication models of *StCOL* genes

**FIGURE 4:**
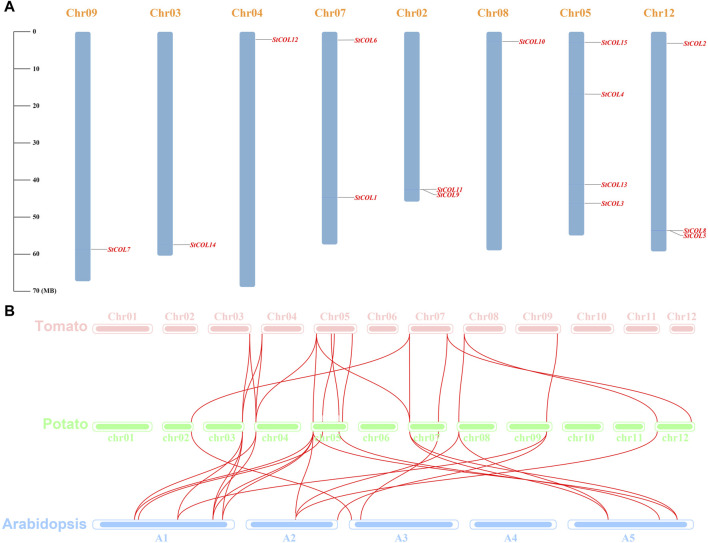
Chromosome location and synteny analysis. **(A)** Chromosome location of potato *StCOL* genes on the chromosome. **(B)** Duplication genes and collinearity orthologs were mapped to each chromosome among potato, tomato, and *Arabidopsis*.

**FIGURE 5:**
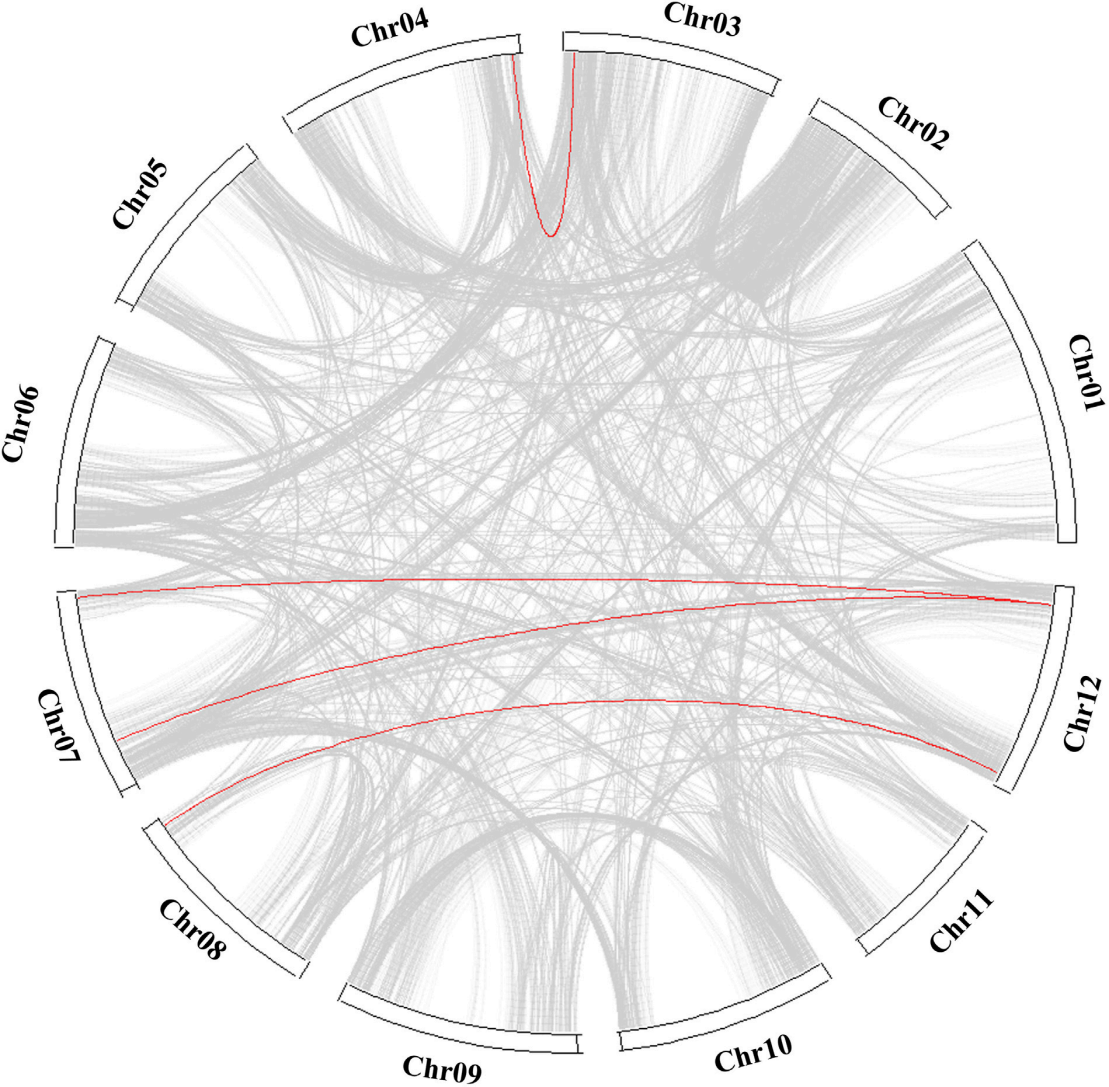
Collinearity analysis of *COL* genes between tomato, potato, and *Arabidopsis*. The red line represents collinearity between *StCOL* genes across species.

Fifteen *COL* genes were found to be unequally distributed on eight potato chromosomes through chromosome localization (Figure 4A). Chr5 has the largest distribution with four *COL* genes. Chr3, Chr4, Chr8, and Chr9 contain only one *COL* gene. *StCOLs* were mainly distributed at the top or end of chromosomes. MCScanX was used to analyze the evolution and function of 15 *StCOLs* in the potato chromosome genome. The results showed that four pairs of the *StCOL* gene family had gene duplication events (Figure 5). There were no tandem repeats and large fragment replication, including *StCOL14* on Chr03, *StCOL12* on Chr12, *StCOL6* and *StCOL1* on Chr07, and *StCOL8* gene clusters on Chr12. There were gene duplication events in *StCOL5* and *StCOL8* on Chr12. Interspecies collinearity analysis of *COL* genes of tomato, potato, and *Arabidopsis* identified 17 homologous genes between tomato and potato and 21 homologous genes between potato and *Arabidopsis*, indicating that there were more direct homologous genes, close relatives, and similar gene functions among these species (Figure 4B). A total of 13 *StCOL* genes are homologous in tomato and *Arabidopsis* (Supplementary Table S5), indicating that these *StCOL* genes may have a common ancestor in different species that evolved from the same ancestor.

### 3.7 Expression patterns of *StCOL* genes in different potato tissues

**FIGURE 6:**
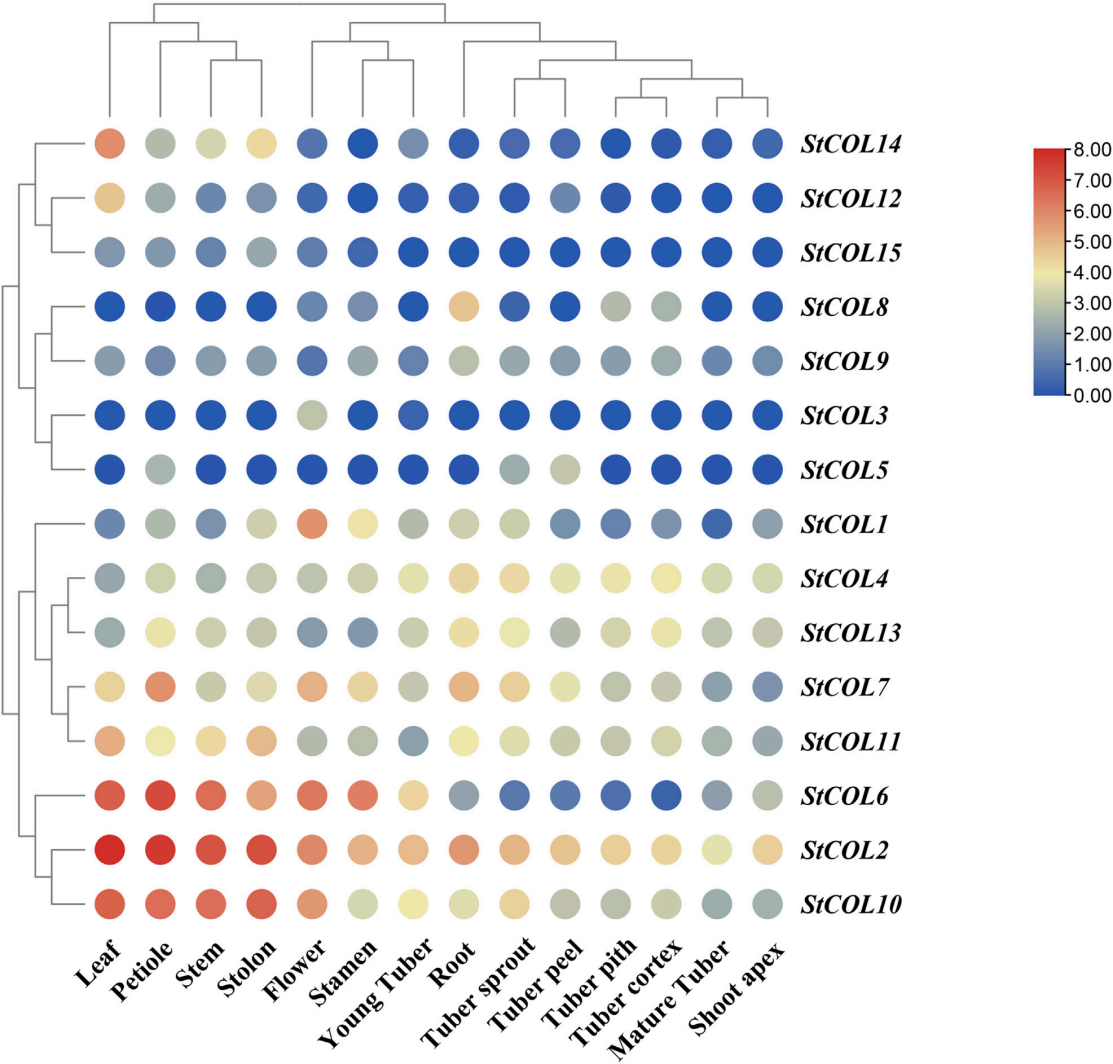
Expression analysis of genes in different tissues by transcriptome data. The horizontal axis represents different tissues in potato, and the vertical axis represents different *StCOL* genes in potato.

The expression patterns of *StCOL* family genes in different potato tissues were analyzed using potato transcriptome data. The results showed that 10 genes were up-expressed to a certain extent in all tissues of potato, while the remaining five genes were down-expressed in potato (Figure 6). *StCOL2* is highly expressed in root, tuber pith, stem, and other tissues, indicating that these genes play an important role in potato growth and development. High expression levels of *StCOL1*, *StCOL2*, *StCOL6*, *StCOL7*, and *StCOL10* were found in flowers and may play a role in regulating the flowering process in potato. The *StCOL2*, *StCOL4*, *StCOL7*, *StCOL10*, *StCOL11*, and *StCOL13* were more highly expressed in root, tuber pith, tuber cortex, and young tuber tissues than other genes. It may be that these genes play a major role in the formation of potato tubers.

### 3.8 Expression analysis of the *StCOL* gene at different maturities of potato leaves and tubers

**FIGURE 7:**
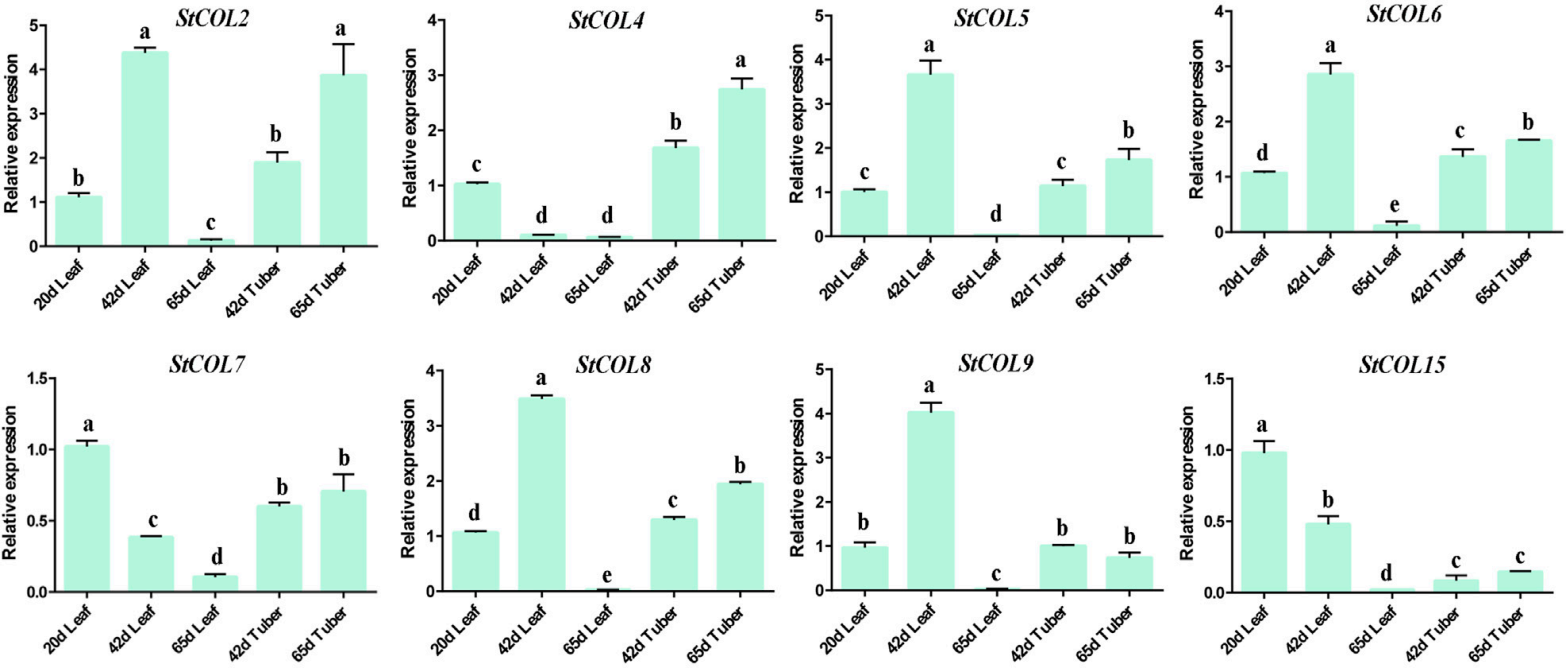
Relative expressions of *StCOL* genes in leaves (20 days, 42 days, and 65 days) and tuber (42 days and 65 days). Duncan’s test with *p* < 0.05 was used to indicate significant differences.

The relative expression levels of *StCOL* genes were measured at different maturities of potato leaves and tubers. The qRT-PCR results showed that the *StCOL2*, *StCOL5, StCOL6*, *StCOL8,* and *StCOL9* were highly expressed in the leaves of potato growing to day 42 (Figure 7). The expression levels of *StCOL4* were significantly higher in potato tubers than in leaves. With increased growth time, the expression level of *StCOL7* and *StCOL15* decreased in leaves and tubers. The expression levels of *StCOL2*, *StCOL6*, and *StCOL9* presented opposite patterns in leaves and tuber during the same period. These results indicated that the *StCOL* genes may play an important role in the formation of potato tubers.

### 3.9 Expression analysis of the *StCOL* gene after different cold stress treatment times

**FIGURE 8:**
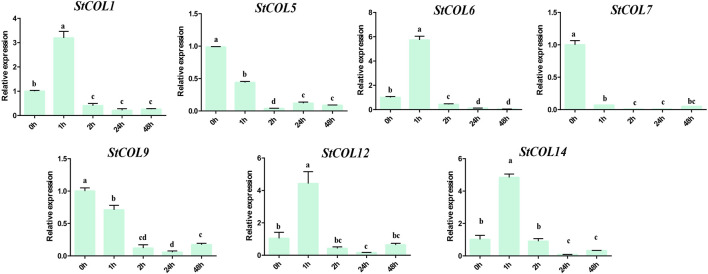
The expression levels of *StCOL* genes at 0 h, 1 h, 2 h, 24 h, and 48 h after 2°C cold treatment. Duncan’s test with *p* < 0.05 was used to indicate significant differences.

The relative expression levels of *StCOL* genes were measured after different cold stress treatment times by qRT-PCR. The results showed that the *StCOL1*, *StCOL6*, *StCOL12*, and *StCOL14* were highly expressed after treatment with 2°C for 1 h (Figure 8). Compared with 0 h, the expression levels of *StCOL5*, *StCOL7*, and *StCOL9* were significantly down-expressed after different lengths of cold stress treatment times. These results indicated that the *StCOL* genes may play an important role in potato response to cold stress.

## 4 Discussion

Potato is an important food crop worldwide. Potato tubers are rich in nutrients, including starch, protein, and vitamin C. The tuberization of potato varies by geographic region and harvest time. Low-temperature stress seriously affected the growth of potato leaves in the aboveground part and the formation of potato blocks in the underground part. Many studies have indicated that the *StCO* gene plays an important role in regulating potato formation (González-Schain et al., 2012; Abelenda et al., 2016). Although the genome-wide identification, characterization, and expression profiling of the *COL* gene family have been reported in potato (Li et al., 2023), the molecular mechanism of the potato COL family involved in the regulation of potato tuberization and response to low-temperature stress is still unclear. In this study, a total of 15 *StCOL* genes were identified, and the expression patterns of these genes at different maturities of potato leaves and tubers and different cold-stress treatment times were determined.

The *CO* genes play a vital role in the flowering process of plants. Under long-day conditions, the DELLA protein directly interacts with CO to regulate flowering in *Arabidopsis* (Xu et al., 2016). Previous studies have shown that *Arabidopsis thaliana*, as a tolerant long-day (LD) plant, specifically accumulates *FT* transcripts in LD conditions, and activation of *FT* transcription is regulated by CCT factor CONSTANS (*AtCO*) (An et al., 2004). In rice, *Hd1* transcripts are similar to *AtCO*, and the gene activity of *Hd1* is mediated by light-activated photopigments because rice *se5* mutants with impaired chromophore biosynthesis exhibit a severe early flowering phenotype in LD conditions, and the *FT* homologous gene *Hd3a* in rice also regulates flowering (Yano et al., 2000; Hecht et al., 2005). The expression of *OsCOL9* enhances rice disease resistance by enhancing the expression of plant hormone biosynthesis genes *NPR1*, *WRKY45,* and *OsACO1* (Liu et al., 2016). In SD and LD conditions, overexpression of *OsCOL15* leads to a delayed flowering phenotype (Wu et al., 2018). The *COL* gene also plays an important role in leaf growth and tuber formation, and photoperiod signal perception mainly occurs in leaf organs. Previous studies have shown that nine *PtCOL* genes are widely expressed in various tissues and organs of poplar but are preferentially expressed in leaves (Li et al., 2020). Potato tuber formation requires short-day regulation, and this photoperiod response is related to the activation of the *StSP6A* gene in leaves. Potato *StCOL1* inhibits the formation of storage organs by directly activating the *FT*-*like StSP5G* repressor protein (Abelenda et al., 2016). The relative expression of the *StCOL* gene in leaves and tubers of “Favorita” was detected at different growth stages. The expression levels of *StCOL2*, *StCOL5*, *StCOL6*, *StCOL8*, and *StCOL9* in leaves were significantly higher than those in tubers, suggesting that these genes may play an important role in the development of potato leaves. Moreover, the expression levels of *StCOL4* in potato tubers are higher than those in leaves, and the expression levels increase with growth time. Many studies have found that *StCDF1* represses the expression of *StCO1/2* by binding to the promoter of *StCO1*, *StCO2,* and *StCO3* genes in potato (Kloosterman et al., 2013; Ramírez Gonzales, 2021). The overexpression of *Nelumbo nucifera COL5* in potato led to increased tuber weight and starch content under SD conditions (Cao et al., 2021). The *StCOL* genes identified in this study provide a data basis for further study of *StCOL* gene function in tuber formation.

Low-temperature stress seriously affects the production, development, and geographical distribution of plants (Ding et al., 2020). A total of 15 *COL* genes were identified in petunia, of which six were induced by cold treatment (Khatun et al., 2021). In this study, seven *COL* genes were differentially expressed under 1 h of cold treatment. Previous studies indicate that CO integrated photoperiodic and cold stress signals into the flowering genetic pathways (Jung et al., 2012). The *AtCOL1* and *AtCOR27* are rapidly induced in response to low temperature by a CBF-independent pathway (Mikkelsen and Thomashow, 2009). Five *CaCOL* genes were induced by cold, of which *CaCOL02* and *CaCOL03* were remarkably upregulated under cold stress and downregulated by heat stress (Huang et al., 2022). These reports indicated that *COL* genes played an important role in low-temperature stress. The potato *SP6A,* a homolog of the floral inductor *FLOWERING LOCUS T*, which controls tuber formation and *SP6A* expression, is downregulated under high temperatures, preventing tuberization (Lehretz et al., 2019; Park et al., 2022). However, the genes that regulate potato tuberization at low temperatures have not been reported. In this study, the promoter sequences in four *StCOL* genes (*StCOL2*, *StCOL3*, *StCOL9,* and *StCOL15*) contained cis-acting elements related to low-temperature stress response. The *StCOL9* showed downregulated expression after 1 h of cold treatment by qRT-PCR analysis. These results provide a database for further exploring the molecular mechanism of *StCOL* genes involved in the regulation of tuberization under cold stress in potato.

## 5 Conclusion

In this research, a total of 15 *COL* family genes were identified in the potato genome. These *StCOL* genes were unevenly distributed on eight chromosomes, and the conserved motifs and gene structure positions of 15 *COL* genes were identified. Notably, the *StCOL* genes containing cis-acting elements associated with stress, hormones, and growth and development were identified. Moreover, the expression level of *StCOL* genes was determined in different development tissues after different low-temperature treatment times in potato. These studies suggested that *StCOL* family genes might play an important role in growth development, tuber formation, and cold stress response in potato.

## Data Availability

The original contributions presented in the study are included in the article/Supplementary Material; further inquiries can be directed to the corresponding authors.

## References

[B1] Abelenda J. A. Cruz-Oró E. Franco-Zorrilla J. M. Prat S. (2016). Potato StCONSTANS-like1 suppresses storage organ formation by directly activating the FT-like *StSP5G* repressor. Curr. Biol. 26, 872–881. 10.1016/j.cub.2016.01.066 26972319

[B2] Almada R. Cabrera N. Casaretto J. A. Ruiz-Lara S. Villanueva E. G. (2009). *VvCO* and *VvCOL1*, two *CONSTANS* homologous genes, are regulated during flower induction and dormancy in grapevine buds. Plant Cell Rep. 28, 1193–1203. 10.1007/s00299-009-0720-4 19495771

[B3] An H. Roussot C. Sua´rez-Lo´pez P. Corbesier L. Vincent C. Pineiro M. (2004). CONSTANS acts in the phloem to regulate a systemic signal that induces photoperiodic flowering of *Arabidopsis* . Development 131, 3615–3626. 10.1242/dev.01231 15229176

[B4] Bailey T. L. Mikael B. Buske F. A. Martin F. Grant C. E. Clementi L. (2009). MEME SUITE: tools for motif discovery and searching. Nucleic Acids Res. 37, W202–W208. 10.1093/nar/gkp335 19458158 PMC2703892

[B5] Campoli C. Drosse B. Searle I. Coupland G. Korff M. V. (2012). Functional characterisation of *HvCO1*, the barley (*Hordeum vulgare*) flowering time ortholog of *CONSTANS* . Plant J. 69, 868–880. 10.1111/j.1365-313X.2011.04839.x 22040323

[B6] Cao D. Li Y. Lu S. Wang J. Nan H. Li X. (2015). GmCOL1a and GmCOL1b function as flowering repressors in soybean under long-day conditions. Plant Cell Physiol. 56, 2409–2422. 10.1093/pcp/pcv152 26508522

[B7] Cao D. Lin Z. Huang L. Damaris R. N. Li M. Yang P. (2021). A *CONSTANS-Like* gene of *Nelumbo nucifera* could promote potato tuberization. Planta 253, 65–11. 10.1007/s00425-021-03581-9 33564987

[B8] Chen C. Chen H. Zhang Y. Thomas H. R. Frank M. H. He Y. (2020). TBtools: an integrative toolkit developed for interactive analyses of big biological data. Mol. Plant 13, 1194–1202. 10.1016/j.molp.2020.06.009 32585190

[B9] Crocco C. D. Botto J. F. (2013). BBX proteins in green plants: insights into their evolution, structure, feature and functional diversification. Gene 531, 44–52. 10.1016/j.gene.2013.08.037 23988504

[B10] Datta S. Hettiarachchi G. H. C. M. Deng X. W. Holm M. (2006). *Arabidopsis* CONSTANS-LIKE3 is a positive regulator of red light signaling and root growth. Plant Cell 18, 70–84. 10.1105/tpc.105.038182 16339850 PMC1323485

[B11] Ding Y. L. Shi Y. T. Yang S. H. (2020). Molecular regulation of plant responses to environmental temperatures. Mol. Plant. 13, 544–564. 10.1016/j.molp.2020.02.004 32068158

[B12] González-Schain N. D. Díaz-Mendoza M. Zurczak M. Suárez-López P. (2012). Potato CONSTANS is involved in photoperiodic tuberization in a graft-transmissible manner. Plant J. 70, 678–690. 10.1111/j.1365-313X.2012.04909.x 22260207

[B13] González-Schain N. D. Suárez-López P. (2008). CONSTANS delays flowering and affects tuber yield in potato. Biol. Plant 52, 251–258. 10.1007/s10535-008-0054-z

[B14] Griffiths S. Dunford R. P. Coupland G. Laurie D. A. (2003). The evolution of *CONSTANS*-like gene families in barley, rice, and *Arabidopsis* . Plant Physiol. 131, 1855–1867. 10.1104/pp.102.016188 12692345 PMC166942

[B15] Guo Y. H. Luo C. Liu Y. Liang R. Z. Yu H. X. Lu X. X. (2022). Isolation and functional analysis of two CONSTANS-like 1 genes from mango. Plant Physiol. Biochem. 172, 125–135. 10.1016/j.plaphy.2022.01.010 35065373

[B16] Hall B. G. (2013). Building phylogenetic trees from molecular data with mega. Mol. Biol. Evol. 30, 1229–1235. 10.1093/molbev/mst012 23486614

[B17] Hassidim M. Harir Y. Yakir E. Green R. M. (2009). Over-expression of *CONSTANS LIKE 5* can induce flowering in short-day grown *Arabidopsis* . Planta 230, 481–491. 10.1007/s00425-009-0958-7 19504268

[B18] Hecht V. Foucher F. Ferrandiz C. Macknight R. Navarro C. Morin J. (2005). Conservation of *Arabidopsis* flowering genes in model legumes. Plant Physiol. 137, 1420–1434. 10.1104/pp.104.057018 15778459 PMC1088331

[B19] Huang Z. Bai X. Duan W. Chen B. Chen G. Xu B. (2022). Genome-wide identification and expression profiling of CONSTANS-Like genes in Pepper (*Capsicum annuum*): gaining an insight to their phylogenetic evolution and stress-specific roles. Front. Plant Sci. 13, 828209. 10.3389/fpls.2022.828209 35251098 PMC8892298

[B20] Imaizumi T. Schultz T. F. Harmon F. G. Ho L. A. Kay S. A. (2005). FKF1 F-box protein mediates cyclic degradation of a repressor of *CONSTANS* in *Arabidopsis* . Science 309, 293–297. 10.1126/science.1110586 16002617

[B21] Jung J. Seo P. J. Park C. (2012). The E3 ubiquitin ligase HOS1 regulates *Arabidopsis* flowering by mediating CONSTANS degradation under cold stress. J. Biol. Chem. 287, 43277–43287. 10.1074/jbc.M112.394338 23135282 PMC3527915

[B22] Khatun K. Debnath S. Robin A. H. K. Wai A. H. Nath U. K. Lee D. J. (2021). Genome-wide identification, genomic organization, and expression profiling of the CONSTANS-like (COL) gene family in petunia under multiple stresses. BMC Genomics 22, 727–817. 10.1186/s12864-021-08019-w 34620088 PMC8499527

[B23] Kikuchi R. Kawahigashi H. Oshima M. Ando T. Handa H. (2012). The differential expression of *HvCO9*, a member of the *CONSTANS*-like gene family, contributes to the control of flowering under short-day conditions in barley. J. Exp. Bot. 63, 773–784. 10.1093/jxb/err299 22016423 PMC3254679

[B24] Kinmonth-Schultz H. A. Tong X. Lee J. Song Y. H. Ito S. Kim S. H. (2016). Cool night-time temperatures induce the expression of CONSTANS and FLOWERING LOCUS T to regulate flowering in *Arabidopsis* . New Phytol. 211, 208–224. 10.1111/nph.13883 26856528 PMC4887344

[B25] Kloosterman B. Abelenda J. A. Gomez M. M. Oortwijn M. de Boer J. M. Kowitwanich K. (2013). Naturally occurring allele diversity allows potato cultivation in northern latitudes. Nature 495, 246–250. 10.1038/nature11912 23467094

[B26] Ledger S. Strayer C. Ashton F. Kay S. A. Putterill J. (2001). Analysis of the function of two circadian-regulated *CONSTANS-LIKE* genes. Plant J. 26, 15–22. 10.1046/j.1365-313x.2001.01003.x 11359606

[B27] Lehretz G. G. Sonnewald S. Hornyik C. Corral J. M. Sonnewald U. (2019). Post-transcriptional regulation of *FLOWERING LOCUS T* modulates heat-dependent source-sink development in potato. Curr. Biol. 29, 1614–1624. 10.1016/j.cub.2019.04.027 31056391

[B28] Li J. Gao K. Yang X. Khan W. U. Guo B. Guo T. (2020). Identification and characterization of the *CONSTANS-like* gene family and its expression profiling under light treatment in *Populus* . Int. J. Biol. Macromol. 161, 999–1010. 10.1016/j.ijbiomac.2020.06.056 32531358

[B29] Li R. Li T. Wu X. Yao X. Ai H. Zhang Y. (2023). Genome-wide identification, characterization and expression profiling of the CONSTANS-like genes in potato (*Solanum tuberosum* L.). Genes 14, 1174. 10.3390/genes14061174 37372354 PMC10297873

[B30] Liang R. Z. Luo C. Liu Y. Hu W. L. Guo Y. H. Yu H. X. (2023). Overexpression of two CONSTANS-like 2 (MiCOL2) genes from mango delays flowering and enhances tolerance to abiotic stress in transgenic *Arabidopsis* . Plant Sci. 327, 111541. 10.1016/j.plantsci.2022.111541 36417961

[B31] Liu H. Dong S. Sun D. Liu W. Gu F. Liu Y. (2016). CONSTANS-Like 9 (OsCOL9) interacts with receptor for activated C-Kinase 1(OsRACK1) to regulate blast resistance through salicylic acid and ethylene signaling pathways. Plos One 11, e0166249. 10.1371/journal.pone.0166249 27829023 PMC5102437

[B32] Livak K. J. Schmittgen T. D. (2001). Analysis of relative gene expression data using real-time quantitative PCR and the 2^−ΔΔCT^ Method. Methods 25, 402–408. 10.1006/meth.2001.1262 11846609

[B33] Mikkelsen M. D. Thomashow M. F. (2009). A role for circadian evening elements in cold-regulated gene expression in *Arabidopsis* . Plant J. 60, 328–339. 10.1111/j.1365-313X.2009.03957.x 19566593

[B34] Min J. H. Chung J. S. Lee K. H. Kim C. S. (2015). The CONSTANS-like 4 transcription factor, AtCOL4, positively regulates abiotic stress tolerance through an abscisic acid-dependent manner in *Arabidopsis* . J. Integr. Plant Biol. 57, 313–324. 10.1111/jipb.12246 25073793

[B35] Park J. S. Park S. J. Kwon S. Y. Shin A. Y. Moon K. B. Park J. M. (2022). Temporally distinct regulatory pathways coordinate thermo-responsive storage organ formation in potato. Cell Rep. 38, 110579. 10.1016/j.celrep.2022.110579 35354037

[B36] Qiao X. Li Q. H. Yin H. Qi K. Li L. Wang R. (2019). Gene duplication and evolution in recurring polyploidization-diploidization cycles in plants. Genome Biol. 20, 38. 10.1186/s13059-019-1650-2 30791939 PMC6383267

[B37] Ramírez Gonzales L. Shi L. Bergonzi S. B. Oortwijn M. Franco-Zorrilla J. M. Solano-Tavira R. (2021). Potato CYCLING DOF FACTOR 1 and its lncRNA counterpart StFLORE link tuber development and drought response. Plant J. 105, 855–869. 10.1111/tpj.15093 33220113 PMC7985872

[B38] Robson F. Costa M. M. Hepworth S. R. Vizir I. Pin˜eiro M. Reeves P. H. (2001). Functional importance of conserved domains in the flowering-time gene CONSTANS demonstrated by analysis of mutant alleles and transgenic plants. Plant J. 28, 619–631. 10.1046/j.1365-313x.2001.01163.x 11851908

[B39] Samach A. Onouchi H. Gold S. E. Ditta G. S. Schwarz-Sommer Z. Yanofsky M. F. (2000). Distinct roles of CONSTANS target genes in reproductive development of *Arabidopsis* . Science 288, 1613–1616. 10.1126/science.288.5471.1613 10834834

[B40] Steinbach Y. (2019). The *Arabidopsis thaliana* CONSTANS-LIKE 4 (COL4)-a modulator of flowering time. Front. Plant Sci. 10, 651. 10.3389/fpls.2019.00651 31191575 PMC6546890

[B41] Turck F. Fornara F. Coupland G. (2008). Regulation and identity of florigen: FLOWERING LOCUS T moves center stage. Annu. Rev. Plant Biol. 59, 573–594. 10.1146/annurev.arplant.59.032607.092755 18444908

[B42] Wang Y. Tang H. Debarry J. D. Tan X. Li J. Wang X. (2012). *MCScanX*: a toolkit for detection and evolutionary analysis of gene synteny and collinearity. Nucleic Acids Res. 40, e49. 10.1093/nar/gkr1293 22217600 PMC3326336

[B43] Wu W. Zhang Y. Zhang M. Zhan X. Shen X. Yu P. (2018). The rice CONSTANS-like protein OsCOL15 suppresses flowering by promoting Ghd7 and repressing RID1. Biochem. Bioph Res. Co. 495, 1349–1355. 10.1016/j.bbrc.2017.11.095 29154991

[B44] Xu F. Li T. Xu P. B. Li L. Du S. S. Lian H. L. (2016). DELLA proteins physically interact with CONSTANS to regulate flowering under long days in *Arabidopsis* . FEBS Lett. 590, 541–549. 10.1002/1873-3468.12076 26801684

[B45] Yano M. Katayose Y. Ashikari M. Yamanouchi U. Monna L. Fuse T. (2000). *Hd1*, a major photoperiod sensitivity quantitative trait locus in rice, is closely related to the *Arabidopsis* flowering time gene *CONSTANS* . Plant Cell 12, 2473–2484. 10.1105/tpc.12.12.2473 11148291 PMC102231

[B46] Yoo S. K. Chung K. S. Kim J. Lee J. H. Hong S. M. Yoo S. J. (2005). *CONSTANS* activates SUPPRESSOR OF OVEREXPRESSION OF *CONSTANS 1* through *Flowering Locus T* to promote flowering in *Arabidopsis* . Plant Physiol. 139, 770–778. 10.1104/pp.105.066928 16183837 PMC1255994

[B47] Zhang B. Feng M. Zhang J. Song Z. (2023). Involvement of *CONSTANS*-like proteins in plant flowering and abiotic stress response. Int. J. Mol. Sci. 24, 16585. 10.3390/ijms242316585 38068908 PMC10706179

[B48] Zobell O. Coupland G. Reiss B. (2005). The family of CONSTANS-like genes in *Physcomitrella patens* . Plant Biol. 7, 266–275. 10.1055/s-2005-865621 15912446

