# Supplementary material for: Genome-wide identification and expression analysis of the CONSTANS-like family in potato (Solanum tuberosum L.)
Source: Front Genet. 2024 Jul 9;15:1390411. doi: 10.3389/fgene.2024.1390411 (PMC11263207; doi:10.3389/fgene.2024.1390411)
Supplement: Supplementary file 1 [file Table1.DOCX]

| Gene ID | Stamen | Flower | Mature Tuber | Tuber sprout | Young Tuber | Tuber pith | Leaf | Tuber peel | Shoot apex | Stem | Stolon | Petiole | Tuber cortex | Root |
| --- | --- | --- | --- | --- | --- | --- | --- | --- | --- | --- | --- | --- | --- | --- |
| StCOL1 | 15.57 | 52.45 | 0.40 | 8.40 | 5.73 | 1.25 | 1.56 | 1.96 | 2.94 | 2.13 | 8.62 | 5.38 | 2.17 | 8.63 |
| StCOL2 | 32.59 | 59.40 | 12.57 | 31.97 | 28.75 | 21.34 | 235.66 | 25.43 | 20.96 | 133.10 | 144.45 | 195.81 | 20.16 | 49.35 |
| StCOL3 | 0.00 | 6.64 | 0.00 | 0.00 | 0.26 | 0.00 | 0.00 | 0.00 | 0.00 | 0.00 | 0.00 | 0.00 | 0.00 | 0.00 |
| StCOL4 | 8.76 | 6.88 | 10.15 | 17.71 | 12.28 | 13.39 | 3.46 | 12.67 | 10.14 | 4.70 | 7.31 | 9.12 | 15.17 | 19.35 |
| StCOL5 | 0.00 | 0.00 | 0.00 | 4.11 | 0.00 | 0.00 | 0.00 | 7.16 | 0.00 | 0.00 | 0.00 | 5.07 | 0.00 | 0.00 |
| StCOL6 | 68.72 | 77.24 | 2.90 | 0.87 | 19.15 | 0.66 | 113.05 | 1.02 | 6.19 | 92.95 | 40.78 | 155.58 | 0.28 | 3.17 |
| StCOL7 | 19.39 | 33.43 | 3.03 | 21.51 | 7.51 | 6.61 | 20.36 | 12.68 | 2.20 | 7.98 | 10.98 | 53.92 | 7.46 | 31.67 |
| StCOL8 | 1.89 | 1.41 | 0.00 | 0.33 | 0.00 | 5.50 | 0.00 | 0.00 | 0.00 | 0.00 | 0.00 | 0.00 | 4.68 | 25.59 |
| StCOL9 | 3.47 | 0.73 | 1.59 | 3.46 | 1.24 | 2.72 | 2.80 | 2.64 | 1.70 | 2.62 | 2.75 | 1.67 | 3.91 | 6.22 |
| StCOL10 | 10.15 | 48.77 | 3.92 | 20.71 | 14.94 | 6.27 | 109.35 | 6.66 | 4.51 | 87.34 | 107.61 | 89.98 | 8.14 | 11.44 |
| StCOL11 | 6.10 | 5.84 | 4.77 | 11.33 | 3.01 | 7.36 | 35.29 | 8.13 | 3.70 | 18.62 | 29.42 | 14.20 | 9.36 | 14.63 |
| StCOL12 | 0.00 | 0.39 | 0.00 | 0.11 | 0.19 | 0.09 | 25.41 | 1.55 | 0.00 | 1.59 | 2.17 | 4.15 | 0.00 | 0.22 |
| StCOL13 | 2.46 | 2.57 | 6.95 | 13.62 | 8.52 | 9.58 | 3.89 | 5.76 | 7.53 | 8.74 | 7.34 | 13.33 | 13.49 | 16.80 |
| StCOL14 | 0.00 | 0.80 | 0.18 | 0.47 | 1.94 | 0.00 | 57.73 | 0.49 | 0.39 | 9.84 | 18.17 | 5.77 | 0.10 | 0.22 |
| StCOL15 | 0.37 | 1.10 | 0.00 | 0.00 | 0.00 | 0.00 | 2.30 | 0.00 | 0.00 | 1.28 | 3.53 | 2.45 | 0.00 | 0.00 |

**Table S1.** The publicly available RNA-seq data of StCOL genes in potato genome

**Table S2.** qRT-PCR primer sequences

| Name | Forward Primer | Reverse Primer |
| --- | --- | --- |
| *StCOL1* | AGGAGTGATGCTGTATTG | CTTACTTCTGCTCTTGTCT |
| *StCOL2* | ACCGCATATTATTCATCAT | TTGTTGTTGTTGTTGTTC |
| *StCOL4* | AAGATACCTAATGCTACTA | TGTCTATCGGATTATGAG |
| *StCOL5* | CTGTTGTTAGATGTGTTGAG | GCATTGATGAACCTGAAC |
| *StCOL6* | AGTAGATGCTTGTATTGGA | TTGTAATGAGGTTGAGATTG |
| *StCOL7* | ATTGAGACGAAGTGCTTA | GGAGGATTATGAGATTGTTC |
| *StCOL8* | CTGTTGTTAGATGTGTTGAG | GCATTGATGAACCTGAAC |
| *StCOL9* | TGATTCTATGATGATTGG | GTTGTTCTTATTGTTGTC |
| *StCOL13* | CGACAATGCTACTTATGG | CATCACTTCTGCTACTTG |
| *StCOL14* | CTGTTGTTGCTTGTCATT | TTACTGCTGCCATTACTT |
| *StCOL15* | CCTTGGACTAATGGTGAA | TTGTAACTCTCGCTTCTC |

**Table S3.**COL gene family identified in potato

| Gene Name | Amino acid (AA) | Molecular weight （kD） | Isoelectric poin （pI) | Instability index （II) | Aliphatic index | Grand average of hydropathicity   (GRAVY) |
| --- | --- | --- | --- | --- | --- | --- |
| *StCOL1* | 411 | 45.67 | 5.41 | 55.92 | 57.18 | -0.593 |
| *StCOL2* | 357 | 39.09 | 5.30 | 49.47 | 62.91 | -0.486 |
| *StCOL3* | 422 | 46.50 | 5.13 | 50.56 | 64.50 | -0.508 |
| *StCOL4* | 453 | 49.92 | 6.09 | 6.94 | 66.56 | -0.600 |
| *StCOL5* | 401 | 44.41 | 5.19 | 59.63 | 60.32 | -0.611 |
| *StCOL6* | 385 | 42.40 | 6.05 | 36.77 | 71.48 | -0.331 |
| *StCOL7* | 379 | 43.06 | 5.78 | 56.76 | 61.48 | -0.792 |
| *StCOL8* | 408 | 45.12 | 5.26 | 59.43 | 59.53 | -0.600 |
| *StCOL9* | 405 | 44.94 | 5.57 | 41.68 | 65.78 | -0.558 |
| *StCOL10* | 347 | 38.65 | 5.30 | 49.85 | 61.04 | -0.595 |
| *StCOL11* | 413 | 45.87 | 5.82 | 43.34 | 63.08 | -0.683 |
| *StCOL12* | 428 | 48.81 | 5.44 | 49.62 | 59.42 | -0.867 |
| *StCOL13* | 413 | 45.05 | 5.61 | 59.96 | 60.24 | -0.512 |
| *StCOL14* | 402 | 45.96 | 5.61 | 50.87 | 64.83 | -0.800 |
| *StCOL15* | 387 | 44.47 | 5.16 | 40.40 | 70.00 | -0.791 |

**Table S4.** Predicted StCOL protein sequence features

| Name | PKC | CK II | cAMP-cGMP | Tyr | N-Myr | N-Glyc |
| --- | --- | --- | --- | --- | --- | --- |
| *StCOL1* | 8 | 8 | 0 | 1 | 2 | 2 |
| *StCOL2* | 6 | 8 | 0 | 0 | 3 | 4 |
| *StCOL3* | 5 | 7 | 2 | 1 | 8 | 5 |
| *StCOL4* | 10 | 9 | 0 | 0 | 7 | 4 |
| *StCOL5* | 8 | 7 | 1 | 1 | 4 | 2 |
| *StCOL6* | 4 | 8 | 0 | 0 | 5 | 3 |
| *StCOL7* | 3 | 6 | 1 | 0 | 2 | 2 |
| *StCOL8* | 8 | 7 | 1 | 1 | 6 | 2 |
| *StCOL9* | 4 | 7 | 0 | 0 | 4 | 1 |
| *StCOL10* | 5 | 8 | 0 | 0 | 2 | 1 |
| *StCOL11* | 2 | 8 | 0 | 0 | 6 | 0 |
| *StCOL12* | 8 | 11 | 1 | 0 | 7 | 0 |
| *StCOL13* | 7 | 8 | 1 | 1 | 10 | 3 |
| *StCOL14* | 8 | 4 | 2 | 0 | 6 | 3 |
| *StCOL15* | 6 | 9 | 2 | 0 | 3 | 4 |

**Table S5** Collinearity analysis of COL genes in poato, tomato and *Arabidopsis*

| ID1 | ID2 | E-value | COL_id1 | COL_id2 |
| --- | --- | --- | --- | --- |
| Solyc03g119540 | Soltu.DM.03G034030.1 | 0 | SlCOL16b | StCOL14 |
| Solyc03g119540 | Soltu.DM.04G002000.1 | 5.00E-94 | SlCOL16b | StCOL12 |
| Solyc04g007210 | Soltu.DM.03G034030.1 | 1.00E-99 | SlCOL16a | StCOL14 |
| Solyc04g007210 | Soltu.DM.04G002000.1 | 0 | SlCOL16a | StCOL12 |
| Solyc05g009310 | Soltu.DM.04G002000.1 | 4.00E-153 | SlCOL16c | StCOL12 |
| Solyc05g046040 | Soltu.DM.05G018860.1 | 0 | SlCOL12 | StCOL3 |
| Solyc05g009310 | Soltu.DM.05G003170.1 | 0 | SlCOL16c | StCOL15 |
| Solyc05g024010 | Soltu.DM.05G012520.1 | 0 | SlCOL14 | StCOL4 |
| Solyc05g020020 | Soltu.DM.05G017200.1 | 0 | SlCOL10b | StCOL13 |
| Solyc05g009310 | Soltu.DM.07G001900.1 | 1.00E-10 | SlCOL16c | StCOL6 |
| Solyc07g006630 | Soltu.DM.02G030260.1 | 1.00E-74 | SlCOL | StCOL11 |
| Solyc07g006630 | Soltu.DM.07G001900.1 | 0 | SlCOL | StCOL6 |
| Solyc07g045180 | Soltu.DM.07G014960.1 | 0 | SlCOL10a | StCOL1 |
| Solyc07g045180 | Soltu.DM.12G023800.1 | 4.00E-177 | SlCOL10a | StCOL8 |
| Solyc08g006530 | Soltu.DM.08G002010.1 | 0 | SlCOL4b | StCOL10 |
| Solyc08g006530 | Soltu.DM.12G003910.1 | 2.00E-152 | SlCOL4b | StCOL2 |
| Solyc09g074560 | Soltu.DM.09G022940.1 | 0 | SlCOL13 | StCOL7 |
| AT1G73870 | Soltu.DM.03G034030.1 | 1.00E-78 | AtCOL7 | StCOL14 |
| AT1G68520 | Soltu.DM.03G034030.1 | 7.00E-93 | AtCOL6 | StCOL14 |
| AT1G49130 | Soltu.DM.03G034030.1 | 3.00E-24 | AtCOL8 | StCOL14 |
| AT1G68520 | Soltu.DM.04G002000.1 | 4.00E-95 | AtCOL6 | StCOL12 |
| AT1G25440 | Soltu.DM.04G002000.1 | 7.00E-98 | AtCOL16 | StCOL12 |
| AT1G68520 | Soltu.DM.05G003170.1 | 5.00E-88 | AtCOL6 | StCOL15 |
| AT1G28050 | Soltu.DM.05G012520.1 | 4.00E-103 | AtCOL15 | StCOL4 |
| AT1G25440 | Soltu.DM.05G003170.1 | 3.00E-100 | AtCOL16 | StCOL15 |
| AT1G73870 | Soltu.DM.05G003170.1 | 2.00E-70 | AtCOL7 | StCOL15 |
| AT1G49130 | Soltu.DM.09G022940.1 | 4.00E-10 | AtCOL8 | StCOL7 |
| AT2G24790 | Soltu.DM.05G003170.1 | 1.00E-11 | AtCOL3 | StCOL15 |
| AT2G24790 | Soltu.DM.08G002010.1 | 7.00E-84 | AtCOL3 | StCOL10 |
| AT2G47890 | Soltu.DM.09G022940.1 | 4.00E-93 | AtCOL13 | StCOL7 |
| AT2G24790 | Soltu.DM.12G003910.1 | 3.00E-87 | AtCOL3 | StCOL2 |
| AT3G02380 | Soltu.DM.02G030260.1 | 5.00E-121 | AtCOL2 | StCOL11 |
| AT3G07650 | Soltu.DM.07G014960.1 | 1.00E-121 | AtCOL9 | StCOL1 |
| AT5G48250 | Soltu.DM.05G017200.1 | 4.00E-122 | AtCOL10 | StCOL13 |
| AT5G57660 | Soltu.DM.05G003170.1 | 7.00E-12 | AtCOL5 | StCOL15 |
| AT5G24930 | Soltu.DM.07G001900.1 | 2.00E-85 | AtCOL4 | StCOL6 |
| AT5G57660 | Soltu.DM.07G001900.1 | 1.00E-97 | AtCOL5 | StCOL6 |
| AT5G24930 | Soltu.DM.08G002010.1 | 1.00E-93 | AtCOL4 | StCOL10 |
